# Binding induced conformational changes of proteins correlate with their intrinsic fluctuations: a case study of antibodies

**DOI:** 10.1186/1472-6807-7-31

**Published:** 2007-05-17

**Authors:** Ozlem Keskin

**Affiliations:** 1 Koc University, Center for Computational Biology and Bioinformatics and College of Engineering, Rumeli Feneri Yolu, 34450 Sariyer, Istanbul, Turkey

## Abstract

**Background:**

How antibodies recognize and bind to antigens can not be totally explained by rigid shape and electrostatic complimentarity models. Alternatively, pre-existing equilibrium hypothesis states that the native state of an antibody is not defined by a single rigid conformation but instead with an ensemble of similar conformations that co-exist at equilibrium. Antigens bind to one of the preferred conformations making this conformation more abundant shifting the equilibrium.

**Results:**

Here, two antibodies, a germline antibody of 36–65 Fab and a monoclonal antibody, SPE7 are studied in detail to elucidate the mechanism of antibody-antigen recognition and to understand how a single antibody recognizes different antigens. An elastic network model, Anisotropic Network Model (ANM) is used in the calculations. Pre-existing equilibrium is not restricted to apply to antibodies. Intrinsic fluctuations of eight proteins, from different classes of proteins, such as enzymes, binding and transport proteins are investigated to test the suitability of the method. The intrinsic fluctuations are compared with the experimentally observed ligand induced conformational changes of these proteins. The results show that the intrinsic fluctuations obtained by theoretical methods correlate with structural changes observed when a ligand is bound to the protein. The decomposition of the total fluctuations serves to identify the different individual modes of motion, ranging from the most cooperative ones involving the overall structure, to the most localized ones.

**Conclusion:**

Results suggest that the pre-equilibrium concept holds for antibodies and the promiscuity of antibodies can also be explained this hypothesis: a limited number of conformational states driven by intrinsic motions of an antibody might be adequate to bind to different antigens.

## Background

Motions induced by protein-ligand interactions are controlled by the global motions of the proteins, including enzymes and antibody-antigens [1-12]. Elucidation of the mechanisms by which the proteins bind to each other or to ligands is of great importance to control and alter protein associations. Several different models have attempted to explain protein binding mechanisms. The specific action of an enzyme with a single substrate was first explained by the lock and key analogy postulated in the nineteenth century. In this analogy, the lock is the enzyme and the key is the substrate. Only the correctly sized key (substrate) fits into the key hole (active site) of the lock (enzyme). Later, it was realized that not all experimental evidence can be adequately explained by using the lock and key model. Consequently the induced-fit theory, which assumes that the substrate plays a role in determining the final shape of the enzyme and that the enzyme is partially flexible was proposed [13]. This theory explains why certain compounds can bind to the enzyme but do not react: the enzyme has been distorted too much or the ligand is too small to induce the proper alignment and therefore cannot react. Only the proper substrate is capable of inducing the proper alignment of the active site.

Pre-existing equilibrium is another alternative model to describe the mechanisms of protein interactions [14-19]. In this model, a protein native state is defined as an ensemble of closely related conformations that co-exist in equilibrium at its binding site. The ligand will bind selectively to an active conformation, thereby biasing the equilibrium toward the binding conformation. In the pre-existing equilibrium model, one protein adapts multiple structures and, thereby, multiple active-sites and functions. Experimental evidences can increase our understanding of the model. In a recent study, pre-existence of collective dynamics of an enzyme (prolyl cis-trans isomerase cyclophilin A, CypA) was observed. Pre-sampling of conformational substates occurs before the enzyme starts its catalytic function [20]. Another example is the aminoglycoside kinase, in which two sub-sites are formed by the motion of a flexible active-site loop [21]. The isomerization of a tyrosine side-chain was found to be critical in the trypanosomal trans-sialidase; it allows the enzyme to have two isomers, with two distinct active-site configurations and thereby two different activities (glycosyl hydrolase and transferase) [22].

**Figure 1:**
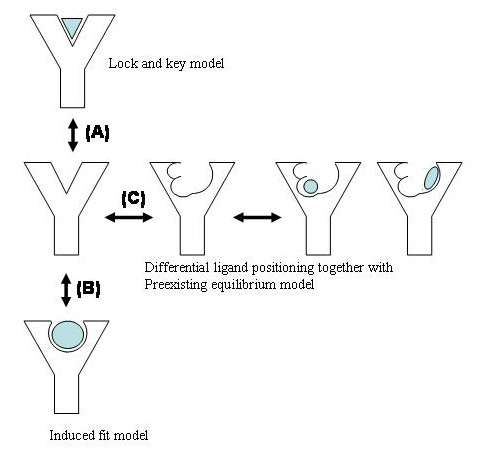
A schematic representation of multispecific ligand recognition by antibodies. (A) Rigid adaptation, (B) Induced fit model, (C) Differential ligand positioning with preexisting equilibrium model

Similarly, antibody-antigen assemblies form an important class of protein complexes exhibiting conformational changes. Antibodies have a limited repertoire of structures that may respond to any incoming antigen without having been previously exposed to it. Yet, antibodies are believed to recognize a practically infinite array of antigens. Thus, a single antibody from this limited repertoire is believed to bind to multiple antigens [23]. The intrinsic conformational flexibility of the antibodies was suggested to facilitate their binding to multiple antigens. This mechanism was supported by thermodynamic data [24]. A similar structural plasticity for the binding site of the T cell receptor (TCR), governing its interaction with the cognate peptide-MHC complex, has also been suggested [25,26]. However, while the existence of flexible antigen-combining sites has been widely recognized, the role of conformational flexibility in the adaptive immune system has not yet been structurally elucidated. In short, classically, antibodies are thought to recognize the antigens through rigid adaptation. An essentially rigid receptor binding site recognizes structurally distinct ligands, without the need for substantial conformational changes in the receptor (Fig 1A). Induced fit model can be an alternative addressing the conformational changes that takes place in the antibodies; an antigen can induce conformational changes in the binding region upon binding (Fig 1B). Antibodies are also thought to recognize the antigens through conformational diversity. A conformationally flexible receptor binding site exists in dynamic equilibrium between different conformational states (pre-existing equilibrium states). Each conformation generates a distinct binding site topology, allowing the receptor to engage multiple ligands at the same region of the binding site (Fig 1C). Salunke and colleagues [26] recently proposed a new mechanism, "differential ligand positioning" for expanding the primary antibody repertoire. The essential feature of this mechanism is that a single antibody conformer may bind diverse antigens at spatially distinct regions of the binding site (Fig 1C). Sethi et al. [26] analyzed a germline monoclonal antibody, 36–65 Fab, which was initially identified in the context of an immune response against the hapten p-azophenylarsonate (Ars). They compared the complexes formed by different peptides and found that conformational flexibility is one of the key features explaining how an antibody binds to different antigens at different regions of the same binding site. Similarly, kinetic analyses and crystallographic studies show that a monoclonal antibody, SPE7 can adopt radically different binding-site conformations and thereby bind to multiple, unrelated antigens [16].

Computationally, Krebs et al. analyzed a set of different proteins with different binding mechanisms by applying normal mode analysis [27]. They found that half of the proteins studied undergo conformational changes that are governed by the two or three lowest modes of the protein. Such results strongly suggest that protein movements between unbound and bound (to a ligand) structures are under selective pressure, so as to follow the lowest frequency normal modes of the protein [28]. Flexibility of a protein allows it to adopt new conformations and, in turn, bind to distinct ligands. This ability of proteins to adopt multiple structures allows functional diversity without depending on the evolution of sequence diversity, and it can greatly facilitate the potential for rapidly evolving new functions and structures. Recently, Liu et al. found that the conformational ensemble of native conditions is determined by the network of cooperative interactions within the protein and suggested that proteins have evolved to use these conformational fluctuations in carrying out their functions [29]. We have previously shown that Anisotropic Network Model (ANM) can be used to establish, *a priori*, the most likely conformational (transitional) changes of an enzyme starting from its unbound state to its three different bound states [30]. In addition, we have shown that the degree of flexibility of the protein is important for proteins to interact with other proteins and as the species gets more complex its proteins become more flexible [31]. Tobi and Bahar [11] successfully showed recently that conformational changes due to protein-protein interactions can be analyzed by the pre-existing equilibrium concept. They applied Gaussian Network Model (GNM) and ANM to study the collective motions of four proteins and indicated that motions calculated from the monomers correlate well with the experimentally observed changes upon complex formation with other proteins. Gu and Bourne's method uses GNM to identify local fluctuation changes important for protein function and residue contacts that contributes to these changes [32].

In this paper, previous studies are extended to investigate the multi-specificity of proteins, especially antibodies. A diverse set of proteins is analyzed with the analytical ANM to study the fluctuations of macromolecules on a coarse grained level. This also allowed us to test the suitability of the method. Pathways between experimentally known conformational changes of a macromolecule upon ligand binding are analyzed based on the pre-existing equilibrium concept for different classes of proteins, i.e. enzymes, binding and transport proteins and antibodies. The last set is studied in detail to elucidate the mechanism of antibody-antigen recognition and binding. The results show that the intrinsic fluctuations obtained by ANM correlate well with structural changes observed when a ligand is bound to the free (unbound) conformation of the protein supporting the pre-existing equilibrium concept. The decomposition of the total fluctuations serves to identify the different individual modes of motion, ranging from the most cooperative ones involving the overall structure, to the most localized ones, manifested as high-frequency fluctuations of individual residues.

It is shown that, ANM is able to find the conformational changes due to ligand binding starting from the unbound form. This suggests that the intrinsic motions of antibodies as well as the electrostatic properties of the binding site that characterize the bound form are sufficiently preserved in the unbound conformation of antibodies. Thus conformational changes of residues that are involved in binding or that are critical for binding can be identified by our method in most cases, starting from the unbound conformation.

## Results

### Comparison of the global mode motions with the conformational changes upon ligand binding

Normal mode analysis has been used to study the collective motions of biological macromolecules. And, some of these normal modes of several proteins are strongly correlated with the large amplitude conformational changes of these proteins observed upon ligand binding [8,10,11,33]

**Figure 2:**
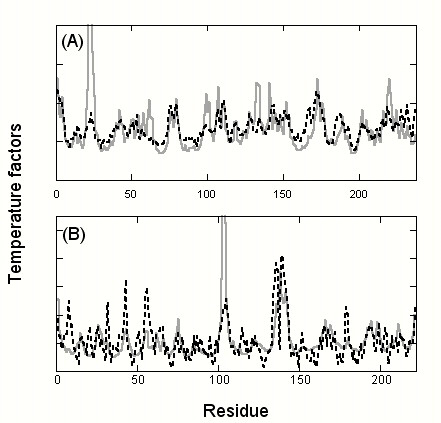
Temperature factors of (A) unbound-LAO (PDB 2lao) and (B) unbound antibody- heavy chain (PDB 2a6j). The dashed lines represent the results of our calculations; solid lines are the experimental temperature factors. Theoretical temperature factors are obtained by using all the collective modes of the proteins. The residues 137–140 are not shown for 2a6j.

Fig 2 compares the residue overall fluctuations obtained from the theoretical and experimental methods for **(A) **K-, R-, Orn-Binding Protein (2lao) and **(B) **antibody heavy chain (2a6j, chain H). Solid and dashed curves represent the experimental and calculated results, respectively. The theoretical values are obtained by the weighted average of the mean square fluctuations over 3N-6 modes of ANM calculations; whereas the experimental data are the x-ray crystallographic temperature factors of individual α-carbons. The agreement between the theory and experiment validates the suitability of the method as discussed in earlier works [6,34-36]. Calculations performed for the other proteins in Table 1 similarly yield results in good agreement with experimental data as discussed below.

Further, the atomic root mean square fluctuations and thus motions from individual modes, obtained by ANM are compared with the experimentally known conformational changes for the list of proteins given in Table 1. C^α ^atomic coordinates for the unbound and bound crystallographic structures are obtained from the PDB. The two structures (unbound and bound) are superimposed (by using their α-carbons) to calculate the individual residue displacements (Δ**R **= **R**_o _- **R**_c_) where **R**_o _- **R**_c _are the crystallographic coordinates of the unbound and bound structures, respectively. The theoretical atomic fluctuations of the unbound structures are obtained with Eq. 5.

**Figure 3:**
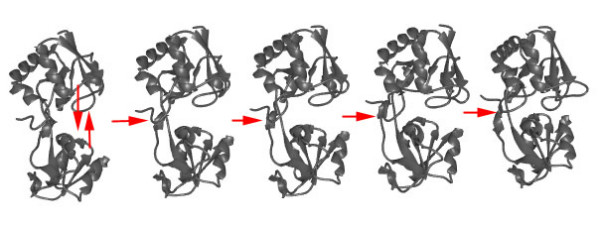
The pathway followed by the for LAO-binding Protein. The left most and right most structures are the unbound and bound conformations, respectively. The intermediate structures are obtained by adding the second global eigenvector to the original coordinates. Since the eigenvectors are normalized to one, a scaling factor of 40, 50 and 60 are used to obtain the intermediate structures. The rmsd values of the intermediate structures are 2.68 (2.55), 2.33 (3.18) and 2.13 (3.82) from the bound (unbound) structure. The two domains of the proteins close as the ligand is bound to the protein.

The results of the comparison of individual mode motions with the real displacements upon ligand binding are presented in Table 2. Mode motions are the mean square residue fluctuations derived from the one of the most global modes (Eq. 5). Theoretical fluctuations give the magnitude of the fluctuations for each residue in the corresponding global modes. Here, the first column gives the mode numbers of the collective motions. We concentrated only on the first three modes, since the slowest modes weigh almost 85% of the all motions [6,35]. The numbers presented in Table 2 give the correlation coefficients between the mean square residue fluctuations for the first three global modes with those of the observed displacements upon ligand binding, separately. In all of the cases, the correlation coefficients are around 0.75. For example, the second mode exhibits a high correlation with the experimentally observed structural changes between the bound and unbound conformers (a correlation coefficient of 0.84) of LAO. The high correlation between the two data indicates that this mode is informative about the conformational changes that take place when a Lys (as a ligand) is bound to the structure. The lowest correlation is for 1ctr with a correlation coefficient 0.63. Even in this case, when one looks at the two curves visually, the similarities are clear (data not shown). The differences between the two curves point to additional local rearrangements upon ligand binding. Fig 3 illustrates the fluctuating conformations visited by the action of the second dominant mode of motions for K-, R-, Orn-Binding Protein. The intermediate structures are obtained by adding the second global eigenvector to the original coordinates. Since the eigenvectors are normalized, a scaling factor of 40, 50 and 60 are used to obtain the intermediate structures. The rmsd values of the intermediate structures are 2.68 Å, 2.33 Å and 2.13 Å compared to the final bound structure, whereas the rmsd values from the initial (unbound) structure increases from 2.55 Å to 3.18 Å to 3.82 Å, respectively.

The results show that there are very high correspondences between the experimental X-ray conformational transitions and the theoretical values. These suggest that among many possible global modes driven by ANM, one or combinations of few global modes can be used to predict the directions of motions of unbound proteins when different ligands are bound. The range of functions that is in our list is very broad, from enzymes to signal transduction proteins. Note that transitions from unbound to bound conformations are mostly represented by the first, second, and third eigenvectors. We might speculate that the specific bound conformations of the proteins should be determined by the specific ligands bound to the unligated structure. And these bound forms should be among the possible conformations that the unbound structure can assume. The ligand interactions drive the structure to a new stable, functional structure among the possible conformations. These results further suggest that the structures assumed by ligand binding in all cases are driven by the pre-existing global fluctuations of the unbound forms. The bound conformation is among the conformations that the unbound protein may undergo based on its intrinsic fluctuations even in the absence of the ligand. And when there is a proper ligand in the environment, the suitable conformation that would fit the ligand might become populated.

**Figure 4:**
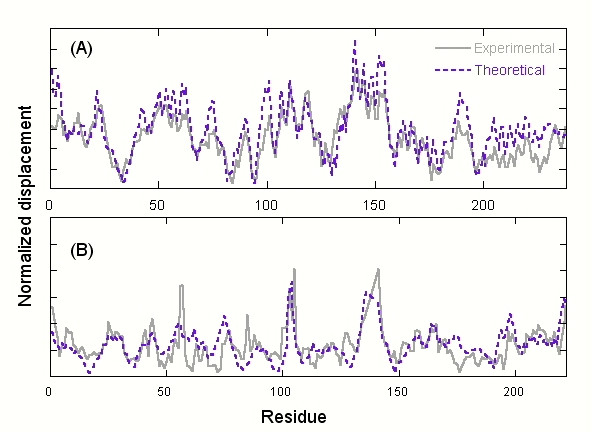
Comparison of theoretical root mean square fluctuations of (A) unbound-LAO (PDB 2lao) and (B) 36–65 Fab, antibody- heavy chain (PDB 2a6j) with the experimentally known conformational changes (ΔR = R_o _- R_c_). The solid curves represent the experimental changes whereas the dashed curves are obtained from the theory. Note that the theoretical curve is scaled to have the same area under the curve with the experimental one. The theoretical fluctuations that are driven by the second and third global modes are shown for LAO and the antibody fragment, respectively.

The results of the cartesian displacements and the theoretically calculated movements are presented in Fig 4(A, B) for the two proteins K-, R-, Orn-binding Protein (2lao-1lst) and antibody heavy chain (2a6j-2a6k). In these figures, the solid curves represent the experimental relative displacements of C^α ^atoms between the bound and unbound forms. The dashed curves are obtained from one of the mean square atomic fluctuations of first three slowest modes of ANM. Note that both the observed and calculated displacements are normalized in these figures. Mairov and Abagyan [37] stated that multidomain proteins may undergo substantial displacements, while their interdomains remain rigid. They studied the stabilized role of ligand in LAO protein. Here the examination of the bound and unbound structures reveals that the LAO undergoes an interdomain motion. Our previous GNM analysis on this protein revealed that it undergoes a domain motion through a hinge axis [36]. The first mode of GNM was responsible for the hinge bending motion constraining the residues F191, G192 and G194 [36]. So, as the first mode of GNM is responsible for the hinge bending motion, the second mode of ANM is representative of the hinge motion and therefore responsible for the ligand driven motion of the protein.

### Antibodies can bind to different antigens

**Figure 5:**
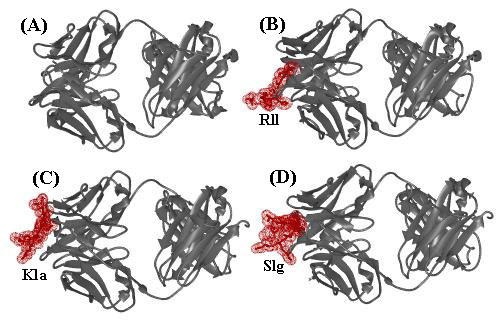
The ribbon diagrams of (A) the unbound 36–65 Fab antibody and (B-D) the 36–65 Fab antibody with three different peptides bound: Rll, RLLIDPPSPRE, KIa, KLASIPTHTSPL, and Slg, SLGDNLTNHNLR, respectively. The peptide atoms are displayed as black spheres. The PDB codes of the unbound antibody is 2a6j. The three PDB codes of the complexes with three different dodecamers are 2a6d, 2a6i and 2a6k.

**Figure 6:**
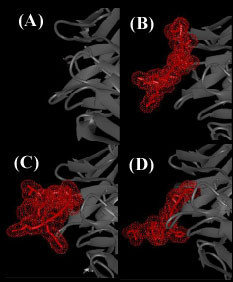
The detailed binding sites of (A) the unbound 36–65 Fab antibody and (B-D) the 36–65 Fab antibody with three different peptides bound: Rll, RLLIDPPSPRE, KIa, KLASIPTHTSPL, and Slg, SLGDNLTNHNLR, respectively.

Recognition of an almost infinite variety of antigens requires the generation of a vast repertoire of antibodies with unique specificities. An antibody bound to an antigen has a different conformation from the same antibody in its unbound form. Both induced fit and pre-existing equilibrium models are observed experimentally to explain the conformational changes between bound and unbound antibodies [15,38,39]. Induced fit suggests that after antigen binds to antibody, interactions take place that convert the initial antibody-antigen complex to a more stable conformation. Pre-existing equilibrium proposes that two or more antibody conformations, one abundant, one rare, exist in equilibrium when not bound to antigen. Antigen binds selectively to the rare conformation, whose free concentration is maintained by re-equilibration from the pool of unbound antibody [40]. Recently, Sethi et al. [26] determined the crystal structures of germline antibody, 36–65 Fab with three structurally diverse dodecapeptides to study the antibody diversity and correlation between the promiscuity of the antibody response and conformational flexibility. Fig 5 shows the ribbon diagrams of the 36–65 Fab antibody **(A) **in its unbound form and **(B) **in complex with RII, a peptide sequence of RLLIDPPSPRE, **(C) **in complex with KIa, a peptide sequence of KLASIPTHTSPL, and **(D) **in complex with Slg of SLGDNLTNHNLR. The PDB code of the unbound antibody is 2a6j. The three PDB codes of the complexes with three different dodecamers are 2a6d, 2a6i and 2a6k. Each dodecamer reveals spatially different footprints in the antigen binding site. Fig 6 displays the binding sites in detail. The crystallographers also find that a single conformational state of the antibody was found to be capable of recognizing these diverse independent dodecamers. Here, the conformational changes (intrinsic motions) of the unbound antibody are investigated with the help of ANM, similar to the cases above. The collective mode motions are compared with the experimental conformational changes that the antibody undergoes when it is bound to the three different dodecamers. Table 3 lists the summary of the results. The first column gives the PDB IDs of the unbound-bound antibodies for three cases. The second column lists the correlation coefficients between the displacements obtained from crystallographic coordinates and theoretical values obtained. The numbers in parentheses show the mode number responsible for the driven motion. In all the cases, the third mode displays the highest correlation with the experimental structural changes thus this mode is believed to be responsible for the antigen driven motions. Therefore, the intrinsic motions obtained from a single mode can drive an antibody to its bound form with different antigens. This is, as stated in Sethi et al. [26] an elegant and simple mechanism for expanding the primary antibody repertoire.

Fig 4B shows the theoretical and experimental conformational changes for the antibody (36–65 Fab). While the overall conformational changes of the unbound structure under the influence of the third mode found by ANM closely resembles the experimental displacements of the antibody, it is important to note that there are minor differences between the two sets of data. Especially, regions ~100–110, 120–130 and 175–200 display slightly different behaviors. The first region corresponds to the H3 loop of the antibody and makes contacts with the antigens. Further this loop is observed to undergo substantial movements between different conformations of the protein. Residues 120–130 connect the variable domain to the constant domain of the antibody.

**Figure 7:**
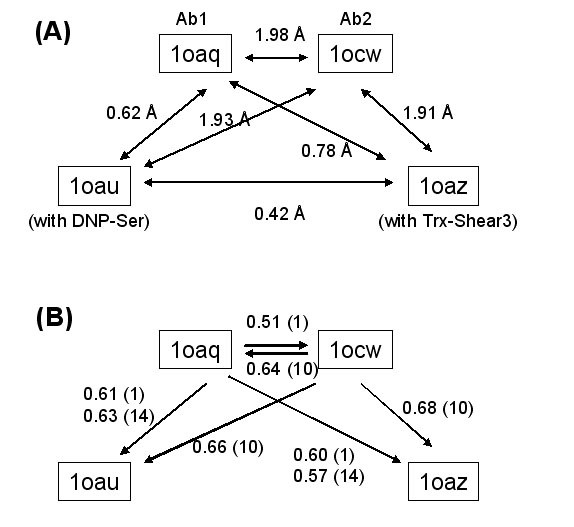
A schematic representation of the similarities of four different conformers of the SPE7 antibody. Part (A) shows the rmsds among the different conformers. Part (B) indicates the most relevant mode to drive the unbound antibodies to the bound ones. The numbers in the parentheses show the responsible mode numbers.

**Figure 8:**
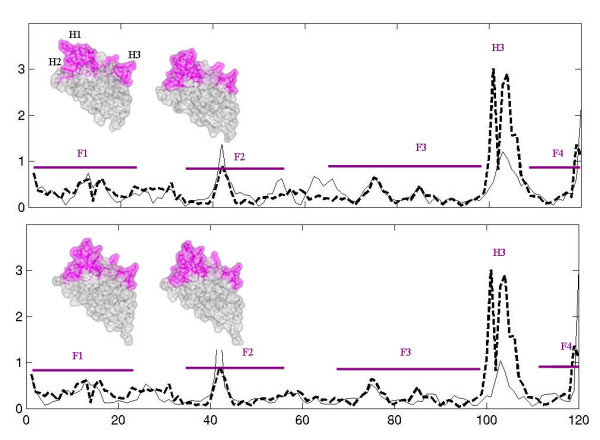
Comparison of global modes with the real displacements for the SPE7 antibody. Dashed lines are for the experimental displacements. Solid lines represent the residue fluctuations for the first and second global modes, respectively (Parts **A **and **B**). The insets are the crystal structures of SPE7. The top structures are for the unbound conformers (1oaq and 1ocw). The lower panel shows the bound conformations (1oau and 1oaz). The purple highlighted regions are the antigen binding regions.

Further, variable region of another antibody, SPE7 is investigated. The crystal structures of the antibody reveals that the same antibody exist in two conformations which can bind to different (structurally distinct) antigens [15]. This antibody puts forward the experimental evidence that unbound antibodies can exist in two conformations, i.e., isomers. The binding region in one conformation is flat with a shallow groove (in Ab1), and binds to recombinant protein, antigen TrxShear3. And the other conformation (Ab2) has a deep hole that binds to aromatic haptens such as DNP. The PDB IDs of the two unbound antibodies are 1ocw and 1aoq. These two unbound structures (which are the variable fragment of the antibody) have a rmsd of 1.98 Å over all alpha carbon atoms. The two bound structures have a hapten (DNP-Ser) and a recombinant protein (Trx-Shear3) as their antigens. The PDB IDs for the bound conformations are 1oau and 1oaz, respectively. Fig 7A shows the rmsd values among the bound and unbound antibodies. The Ab1 antibody (1oaq) is more similar to the Trx-Shear3 bound conformation of the antibody, whereas Ab2 antibody (1ocw) structurally resembles to that of the DNP-Ser bound antibody. When experimental displacements among all conformers of SPE7 variable regions (between unbound forms (Ab1-Ab2) and unbound and bound forms) are compared with the theoretical displacements obtained from individual collective modes, we observe that it is possible to force the proteins from one form to another following the global mode motions. Fig 7B shows the correlation coefficients of the experimental displacements obtained from the crystal coordinates with the theoretical displacements obtained from eigenmodes. Both unbound structures (Ab1 and Ab2) can be converted to the bound conformations following the global motions. Kinetic experiments suggest that an equilibrium between two pre-existing antibody conformers exists, and only one of them (Ab2) binds the ligands. It is difficult to observe this from the analysis herein. Three CDR regions responsible for antigen binding are H1: 26–35, H2: 51–66 and H3: 99–109 as highlighted purple in the antibody structures shown in the insets of Fig 8. The rest of the antibody is labeled as F1 (residues 1–25), F2 (residues 36–50), F3 (residues 67–98) and F4 (residues 110–120). The figure also compares the **(A) **first and **(B) **second global mode fluctuations with the experimental displacements. The three antigen binding loops (H1-H3) and the fragments (F1-F4) are labeled in the figure. As seen from the figure, although the overall correlations are not very high (~0.6), if we concentrate on the fragment regions we observe much better correlations. The first and second modes represent the motions of the antibody fragments of F1-F4 with very high correlations. F1 and F2 are driven by the second mode with a correlation coefficient of 0.81 whereas F3 and F4 are correlated with the first mode with a coefficient of 0.85. The H3 loop of SPE7 is mainly responsible for antigen binding. It is observed that further adjustments are needed for this loop. One of the mentioned collective modes is not enough to represent its motion. The other loops are controlled by the combined effect of the second and first modes (i.e., H1, H2 and H3 experimental transitional changes are correlated with the first and second concerted modes with a coefficient of 0.80, 0.85 and 0.64, respectively).

### One of the global modes is important for conformational changes

Investigation of the collective modes can give us insights about the mechanisms of binding processes. Comparison of the actual experimental conformational changes with the root mean square fluctuations from the collective modes indicates that individual modes are more informative than taking combined effect of all modes. Usually, only *one of the slowest global modes is important to represent the ligand induced conformational changes in proteins*. The results clearly show that the unbound conformation of all the proteins discussed in this study have intrinsic tendencies to reconfigure their conformations into the bound one. Therefore, the mechanism for the recognition and binding of the proteins with their ligands can be estimated a priori, by considering the conformations they undergo under the influence of the collective modes. And the proteins selectively bind to their ligands.

## Discussions and conclusion

It has been shown that for native proteins, the very low frequency normal modes make major contributions to the conformational fluctuations at thermal equilibrium. Such motions can change the interactions of proteins with other molecules and with their environment.

Comparison of the global mode motions with the conformational changes upon ligand binding suggests a high correspondence between the normal mode directions derived from ANM calculations and the actual conformational changes. Here, intrinsic motions of eight proteins, from different classes of proteins, i.e. enzymes, binding and transport proteins and antibodies are examined. The high correlations between the experimental and computational intrinsic motions confirm that the individual eigenvectors might be useful to drive the unbound structure toward bound structures. Thus, the unbound structure can assume a set of conformations driven by the slowest modes, and ligand binding appears to introduce a new stable structure from this set of accessible conformations. The conformational changes exhibited by the proteins cannot fully be explained by the lock and key model or the induced fit model. According to the pre-existing equilibrium hypothesis proteins assume a set of conformations that are related to and in the vicinity of each other.

CheY, LAO-Binding Protein, HPPK (kinase), renin, Thymidylate Synthase are examples that show high correlations (≥ .70) between the experimentally observed structural changes and theoretically predicted conformational motions. Therefore, the unbound conformations of these proteins most probably assume a conformation that strongly resemble their bound forms. On the other hand, aspartyl protease, dihydrofolate reductase, calmodulin show lower correlations (0.63–0.66) which suggest that some further local rearrangements of the structures are needed to reach the bound conformations. These results suggest that pre-existing equilibrium is a key component in the binding process, such that it facilitates in selecting the complex forming conformation among the others but cannot exclusively explain the whole mechanism. Induced fit model should further play a role in the fine tuning of the local arrangements after the ligands are bound.

A limited repertoire of antibodies can recognize and bind to an almost infinite number of antigens. Antibodies are known with their delicate specificity for the antigens they bind; at the same time, a single antibody binding site can accommodate different, if similar, antigens [15]. Kinetic analyses demonstrate that the pre-equilibrium states exist for an antibody to provide specific binding sites accompanied by induced fit. James et al [15] showed that a monoclonal immunoglobulin, SPE7 adopted at least two different conformations that were independent of antigen. Here, the intrinsic motions of a germline antibody of 36–65 Fab with three different peptides is investigated. Similar binding site is used to bind to these three peptides. The third collective mode of the unbound antibody was found to be responsible for the conformational changes undertaken with three different peptide binding. The correlations were around 0.69 for the three cases. This number might suggest that pre-existing equilibrium drives the early binding mechanism, and induced fit model later re-arranges the local arrangements after the ligands bind. The second antibody investigated is antigen binding region of SPE7. Available two pre-existing equilibrium conformations are compared with the two different antigen bound conformers. The results show that the first two modes are responsible for the global motions. The antigen binding loops are further re-arranged under the influence of higher modes. Therefore, our results on a diverse set of proteins show that: proteins undergo intrinsic collective motions driven by their structures, and the intrinsic collective motions of proteins are required for binding and these motions correlate well with the experimental conformational changes and we conclude that these intrinsic collective motions that are driven by the protein structure are required for ligand binding.

## Methods

### Proteins

Seven proteins are analyzed in this study to test the suitability of the method. The crystal structures of both the unbound and bound conformations of these proteins are available in the Protein Data Bank (PDB) [41]. Table 1 lists the proteins analyzed. The unbound and bound form PDB codes are given in the first column. The names of the corresponding proteins, number of residues, root mean square deviations (rmsd) between the unbound and bound forms of the proteins are listed in the following columns of the table. The ligands bound are given in the fourth column of the table. Three antibodies investigated are also listed in the same table.

### Method

The details of the analytical model (ANM) have been given in the Additional file 1 and elsewhere [6,42]. ANM is equivalent to a normal mode analysis. In the model, C^α ^atoms are taken as the interaction sites for residues which are connected by virtual bonds (linear springs between the beads) to form the protein backbone. This model assumes that the protein in the folded state is equivalent to a three dimensional elastic network. Therefore, the molecule is modeled as a chain of N beads (residues) connected by N-1 springs. The bond lengths and bond angles are not constrained, which yields 3N degrees of freedom. The beads are subject to harmonic potentials from all neighboring beads regardless of backbone connections. In the calculations, only interactions between pairwise beads is considered if they are in contact, thus decreasing the spring number from N × (N - 1)/2 to a much smaller number. If the distance between two residues i and j is less than a cutoff distance then these two residues are assumed to be in contact. The cutoff distance includes all residue pairs within a first interaction shell of 15 Å and defines the range of interaction of bonded and non-bonded α carbons. The potential energy equation can be expressed in the matrix representation in terms of the 1 × 3N fluctuation vector (Δ R) and the 3N × 3N Hessian matrix (H) composed of inter-residue force constants as

V=12[ΔR]TH[ΔR]
 MathType@MTEF@5@5@+=feaafiart1ev1aaatCvAUfKttLearuWrP9MDH5MBPbIqV92AaeXatLxBI9gBaebbnrfifHhDYfgasaacH8akY=wiFfYdH8Gipec8Eeeu0xXdbba9frFj0=OqFfea0dXdd9vqai=hGuQ8kuc9pgc9s8qqaq=dirpe0xb9q8qiLsFr0=vr0=vr0dc8meaabaqaciaacaGaaeqabaqabeGadaaakeaacqqGwbGvcqGH9aqpdaWcaaqaaiabigdaXaqaaiabikdaYaaacqGGBbWwcqqHuoarcqqGsbGucqGGDbqxdaahaaWcbeqaaiabdsfaubaakiabbIeaijabcUfaBjabfs5aejabbkfasjabc2faDbaa@3D78@

where the *Hessian *matrix (**H**) is defined as

Hij=∂2V∂ΔRi∂ΔRj
 MathType@MTEF@5@5@+=feaafiart1ev1aaatCvAUfKttLearuWrP9MDH5MBPbIqV92AaeXatLxBI9gBaebbnrfifHhDYfgasaacH8akY=wiFfYdH8Gipec8Eeeu0xXdbba9frFj0=OqFfea0dXdd9vqai=hGuQ8kuc9pgc9s8qqaq=dirpe0xb9q8qiLsFr0=vr0=vr0dc8meaabaqaciaacaGaaeqabaqabeGadaaakeaacqqGibasdaWgaaWcbaGaeeyAaKMaeeOAaOgabeaakiabg2da9maalaaabaGaeyOaIy7aaWbaaSqabeaacqaIYaGmaaGccqqGwbGvaeaacqGHciITcqqHuoarcqqGsbGudaWgaaWcbaGaeeyAaKgabeaakiabgkGi2kabfs5aejabbkfasnaaBaaaleaacqqGQbGAaeqaaaaaaaa@4089@

The equilibrium cross-correlations between residue fluctuations is given by

<Δ*R*_*i *_• Δ*R*_*j*_> = (k_B_T)[H^-1^]_*ij*_

where k_B _is the Boltzmann constant, T is the absolute temperature. The angular brackets designate the vibrations of Cα atoms over all modes of motion, [H^-1^]_ij _is the trace of the 3 × 3 matrix enclosed in the inverse of the 3N × 3N Hessian matrix for residue types i and j. In parallel with normal mode analysis (NMA), the equality in the above equation follows from the fact that the mean square displacements in mode space are inversely proportional to the eigenvalues [34,43-45]. In NMA, the Hessian matrix is mass-weighted, here specificity of the beads is not considered and all residues are assumed to have the same weight. Associated with the normal or fundamental modes of vibration, the inverse of the Hessian matrix can be written as follows over the range of modes 1 ≤ m ≤ 3N-6,

H−1=∑mλm−1[umumT]1≤m≤3N−6
 MathType@MTEF@5@5@+=feaafiart1ev1aaatCvAUfKttLearuWrP9MDH5MBPbIqV92AaeXatLxBI9gBaebbnrfifHhDYfgasaacH8akY=wiFfYdH8Gipec8Eeeu0xXdbba9frFj0=OqFfea0dXdd9vqai=hGuQ8kuc9pgc9s8qqaq=dirpe0xb9q8qiLsFr0=vr0=vr0dc8meaabaqaciaacaGaaeqabaqabeGadaaakeaafaqabeqacaaabaGaeeisaG0aaWbaaSqabeaacqGHsislcqaIXaqmaaGccqGH9aqpdaaeqbqaaGGaciab=T7aSnaaDaaaleaacqWGTbqBaeaacqGHsislcqaIXaqmaaGccqGGBbWwcqWG1bqDdaWgaaWcbaGaemyBa0gabeaakiabdwha1naaDaaaleaacqWGTbqBaeaacqWGubavaaGccqGGDbqxaSqaaiabd2gaTbqab0GaeyyeIuoaaOqaaiabigdaXiabgsMiJkabb2gaTjabgsMiJkabiodaZiabb6eaojabgkHiTiabiAda2aaaaaa@4D2F@

where λ_m _are the eigenvalues of H and u_m _are eigenvectors of H for the mth mode. The six modes with zero eigenvalues correspond to purely translational and purely rotational motion modes. Vibrational modes give the directions and relative amplitudes of the atomic displacements in each mode. [u_m_u_m_^T^] is an 3N × 3N matrix, representing the contribution of each of the mth eigenvectors to H. This equation provides a simple means of decomposing the dynamics into a series of modes. In other words, the mean square fluctuation of residue type i associated with the *m*th mode of motion is found from:

<(ΔRi)2>m=λm−1[um]i[umT]i
 MathType@MTEF@5@5@+=feaafiart1ev1aaatCvAUfKttLearuWrP9MDH5MBPbIqV92AaeXatLxBI9gBaebbnrfifHhDYfgasaacH8akY=wiFfYdH8Gipec8Eeeu0xXdbba9frFj0=OqFfea0dXdd9vqai=hGuQ8kuc9pgc9s8qqaq=dirpe0xb9q8qiLsFr0=vr0=vr0dc8meaabaqaciaacaGaaeqabaqabeGadaaakeaacqGH8aapcqGGOaakcqqHuoarcqWGsbGudaWgaaWcbaGaemyAaKgabeaakiabcMcaPmaaCaaaleqabaGaeGOmaidaaOGaeyOpa4ZaaSbaaSqaaiabd2gaTbqabaGccqGH9aqpiiGacqWF7oaBdaqhaaWcbaGaemyBa0gabaGaeyOeI0IaeGymaedaaOGaei4waSLaemyDau3aaSbaaSqaaiabd2gaTbqabaGccqGGDbqxdaWgaaWcbaGaemyAaKgabeaakiabcUfaBjabdwha1naaDaaaleaacqWGTbqBaeaacqWGubavaaGccqGGDbqxdaWgaaWcbaGaeeyAaKgabeaaaaa@4CE8@

The motions from individual modes are compared with the experimentally known conformational changes for the list of proteins given in Table 1. C^α ^coordinates for the unbound and bound structures are obtained from the PDB. The two structures (unbound and bound) are superimposed to calculate the individual residue displacements

Δ**R**_theor _= **R**_o _- **R**_c_

where **R**_o _- **R**_c _are the crystallographic coordinates of the unbound and bound structures, respectively. The theoretical atomic fluctuations of the unbound structures are obtained with Eq. 5. In previous similar studies, conformational changes have been compared to normal modes through the measure of scalar products. In this study Pearson correlation coefficients are used to have a consistent measure.

## Supplementary Material

Additional file 1 Anisotropic Network Model (ANM). The file contains the details of the method (ANM) used in the study. Click here for file

## Figures and Tables

**Table 1:** List of proteins studied.

Protein ID (unbound-bound)	Name of the protein	Number of residues	Ligand	Rmsd between the structures (Å)
3chy-1chn	CheY	128	Mg	1.39
5dfr-4dfr^	Dihydrofolate Reductase	158	Methotrexate	0.80
1ctr-1cll	Calmodulin	138	Trifluoperazine	14.4
2lao-1lst	K-, R-, Orn-Binding Protein	238	Lys	4.70
3tms-2tsc	Thymidylate Synthase	264	UMP, CB3	0.80
1hka-1rao	HPPK (kinase)	158	AMP	3.13
1bbs-1rne	Renin	326	CGP, NAG	6.16
2a6j-2a6d*	Antibody-Heavy chain	222	Dodecapeptide1	0.94
2a6j-2a6i*	Antibody-Heavy chain	222	Dodecapeptide2	1.10
2a6j-2a6k*	Antibody-Heavy chain	222	Dodecapeptide3	1.00
1oaq-1oau	Antibody-Heavy chain	121	DNP-Ser	0.62
1ocw-1oaz	Antibody-Heavy chain	121	Trx-Shear3	1.91

The first column lists the unbound and bound conformations PDB codes. The second column gives the protein description; the third column gives the residue numbers of the proteins. The fourth column lists the ligand bound to the protein. The last column provides the root mean square deviation (rmsd) between the unbound – bound structures considering the alpha carbons.

^The alignments are calculated over two stretches: 1–15 and 21–159.

* The alignments are calculated over two stretches: 1–136 and 141–222.

**Table 2:** Correlation coefficients between the real displacements and theoretical values

Mode #	3chy	5dfr	1ctr	2lao	3tms	1hka	1bbs
1	0.60	**0.66**	0.47	0.37	0.10	0.72	0.60
2	**0.73**	0.42	0.14	**0.84**	**0.70**	0.85	0.40
3	0.68	0.43	**0.63**	0.23	0.05	**0.87**	**0.71 **(4)

The first column gives the mode numbers, the first row lists the PDB IDs of the unbpund structures. The bold numbers highlights the highest correlation coefficient for the respective protein.

**Table 3:** Correlation coefficients between the real displacements and theoretical values for the antibody bound to three different dodecamers.

PDB ID of the Unbound-Bound Antibody	Correlation coefficient
2a6j-2a6d	0.66 (3)
2a6j-2a6i	0.69 (3)
2a6j-2a6k	0.71 (3)

The first column lists the PDB IDs of the unbound and bound structures of the antibodies. The number in the parentheses correspond to the mode numbers.
