# Supplementary material for: Binding induced conformational changes of proteins correlate with their intrinsic fluctuations: a case study of antibodies
Source: BMC Struct Biol. 2007 May 17;7:31. doi: 10.1186/1472-6807-7-31 (PMC1888692; doi:10.1186/1472-6807-7-31)
Supplement: Additional file 1 — Anisotropic Network Model (ANM). The file contains the details of the method (ANM) used in the study. [file 1472-6807-7-31-S1.doc]

**Anisotropic Network Model (ANM)**

The interaction potential energy between all pairs of interacting residues is assumed to obey a harmonic potential. This corresponds to there being a Gaussian distribution of interatomic distances about their equilibrium values. The coordinates of the protein are used for the equilibrium positions. If k is the force constant of harmonic potential adopted for all residue pairs, neglecting the residue types, then the harmonic potential energy of a protein governing the fluctuations is given by: V_(i,j)_ = ½ k ΔR_(i,j)_^2^.

In general, for small values of displacements (fluctuations), the potential energy will be the sum of the displacements and it may be expressed as a power series in the displacements ΔR_i_ [molecular vibrations]:

In the equation above, by choosing the zero of energy so that the energy of the equilibrium configuration is zero, V_0_ may be eliminated. Furthermore, when all the ΔR’s are zero, the atoms are all in their equilibrium positions so that the energy must be a minimum since in the native state, the protein assumes a conformation that is energy minimized with respect to all residue fluctuations. Therefore, the first derivative which gives the net force acting on the system must be zero.

For sufficiently small amplitudes of vibration, the higher terms (cubic, quadratic, etc. in the ΔRi’s) can be neglected, so that the power series reduces to:

This potential is equivalent to the harmonic potential energy of springs that assumes that the potential energy can be approximated as a sum of quadratic terms in the displacements. The potential energy equation can be expressed in the matrix representation in terms of the 1x3N fluctuation vector (ΔR) and the 3Nx3N Hessian matrix (H) composed of interresidue force constants as

If we define the *Hessian* matrix (**H**) as

The ijth super-element (i ≠ j) **H**ij of **H** is

The equilibrium cross-correlations between residue fluctuations is given by:

where k_B_ is the Boltzmann constant, T is the absolute temperature. The angular brackets designate the vibrations of C^α^ atoms over all modes of motion, [H^-1^]_ij_ is the trace of the 3x3 matrix enclosed in the inverse of the 3Nx3N Hessian matrix for residue types i and j, and d{ΔR} is the integration all over the residue fluctuations. In parallel with normal mode analysis, the last equality in the above equation follows from the fact that the mean square displacements in mode space are inversely proportional to the eigenvalues [44, 46]

It should be noted that the determinant of H is zero. Therefore, H cannot be inverted directly. H^-1^ is an approximation calculated from 3N-6 nonzero eigenvalues, λ_m_, and their corresponding eigenvectors, u_m_, of matrix H. A major problem with full atomic normal mode analysis is the limitations on the computer memory (~(3n)^2^), where n is the number of atoms in the protein. Here we reduced the dimensions of the matrix to 3N where N is the number of residues in the protein, then determined the global motions and flexible regions in a protein. Since the dimensions of the matrix to be inverted is only three times greater than the number of residues in a protein, substantially larger proteins can be considered rather than the conventional atomic molecular dynamics.

**Modal decomposition:**

Information on global dynamics is acquired by decomposing the motions into a series of modes, and concentrating on the modes at the slowest (largest amplitude) end of the spectrum. The latter are the most cooperative modes in the folded state, in that they dominate the coupled collective motions of large size structural blocks. To elucidate the mechanism of these motions, as a first step H can be written as the product of the matrix U, the diagonal matrix Λ, and the transpose of U as:

$H=U\Lambda U^{T}$ = [u_1_ u_2_ u_3_….u_3N_] diag(λ_1_ λ_2_ λ_3_ ………λ_3N_) [u_1_ u_2_ u_3_….u_3N_] ^T^

The diagonal elements of the Λ are the eigenvalues (λ_m_) of H. We note that since U is orthonormal matrix whose columns are eigenvectors (u_m_) of H, U^T^=U^-1^. The inverse of H is found from H^-1^=U Λ^-1^ U^T^ or in other words, as the sum of the contributions from each mode:

1 ≤ m ≤ 3N-6

The above equation is carried out over the range of modes 1 ≤ m ≤3N-6, associated with the normal or fundamental modes of vibration. The first six modes with zero eigenvalues correspond to purely translational and purely rotational motion modes. Vibrational modes give the directions and relative amplitudes of the atomic displacements in each mode. [u_m_u_m_^T^] is an 3Nx3N matrix, representing the contribution of each of the mth eigenvectors to H. This equation provides a simple means of decomposing the dynamics into a series of modes. In other words, the mean square fluctuation of residue type i associated with the mth mode of motion is found from:

[u_m_u_m_^T^]_ii_ is simply found by the product of [u_m_]_i_  with [u_m_^T^]_i_ and the result is a scalar giving the mean square fluctuation of residue type i.

## The cross correlations, *CC(i,j)* between fluctuations of C^α^ atoms are normalized as:

The positive and negative limits of CC(i,j) are (1 and -1) and correspond to pairs of residues exhibiting fully correlated (same direction, same sense) and fully anticorrelated (same direction, opposite sense) motions, respectively.

In this study, the specificity of side chains is not considered, and including the side chains may improve the specificity of the model at a cost of computational time. There have been ongoing projects where different residues are parameterized with different force constants, or according to their hydrophobicity scale.
